# Trajectories of Anxiety and Depression Symptoms over Five Years since Breast Cancer Diagnosis: Results of the NEON-BC Prospective Study

**DOI:** 10.3390/healthcare10040661

**Published:** 2022-03-31

**Authors:** Catarina Lopes, Milton Severo, Filipa Fontes, Luisa Lopes-Conceição, Augusto Ferreira, Susana Pereira, Nuno Lunet, Natália Araújo

**Affiliations:** 1 EPIUnit—Instituto de Saude Publica, Universidade do Porto, Rua das Taipas, 135, 4050-600 Porto, Portugal; catarina.lopes@ispup.up.pt (C.L.); milton@med.up.pt (M.S.); filipa.fontes@ispup.up.pt (F.F.); luisa.conceicao@ispup.up.pt (L.L.-C.); susana.pereira@ipoporto.min-saude.pt (S.P.); nlunet@med.up.pt (N.L.); 2 Laboratorio para a Investigaçao Integrativa e Translacional em Saude Populacional (ITR), Rua das Taipas, 135, 4050-600 Porto, Portugal; 3 Departamento de Ciencias da Saude Publica e Forenses e Educaçao Medica, Faculdade de Medicina da Universidade do Porto, Alameda Prof. Hernâni Monteiro, 4200-319 Porto, Portugal; 4 Instituto Portugues de Oncologia do Porto, R. Dr. António Bernardino de Almeida 865, 4200-072 Porto, Portugal; augusto.carmo.ferreira@ipoporto.min-saude.pt

**Keywords:** breast cancer, anxiety, depression, longitudinal study, trajectory

## Abstract

Anxiety and depression symptoms are frequent among patients with breast cancer (BCa) and may last after initial treatments. We aimed to identify five-year trajectories of anxiety and depression symptoms among women with BCa. Neuro-oncological complications of BCa (NEON-BC) cohort included 506 patients admitted at the Portuguese Institute of Oncology of Porto in 2012, who were evaluated with the Hospital Anxiety and Depression Scale before cancer treatment and after one, three, and five years (7.9% attrition rate). Mixed-effect models were used to model anxiety and depression scores over time and model-based clustering to identify the different trajectories. Three trajectories of anxiety symptoms were identified: (1) high scores at baseline and increasing over time (21.7%); (2) consistently low scores over time (63.6%); (3) mid-range scores at baseline, decreasing over time (14.6%). Three trajectories were identified for depression symptoms: (1) high scores at baseline and increasing over time (21.1%); (2) mid-range scores at baseline, which decreased afterward (58.7%); (3) consistently low levels over time (20.2%). Age, education, baseline, and one-year anxiety/depression status predicted the worst five-year trajectories. These results show that assessing anxiety and depression symptoms before treatment and after one year may contribute to identifying the patients who could benefit the most from psychological support.

## 1. Introduction

Breast cancer is the most prevalent type of cancer among women [1]. The 5-year survival rate reaches nearly 90% in some countries, which can be explained by improvements in access to screening programs, earlier detection, and progress in therapeutic guidelines and treatment [2].

However, this longer survival is sometimes accompanied by physical and psychological complications of cancer and its treatments [3]. Clinical levels of anxiety and depression are estimated to be present in nearly half of patients newly diagnosed with cancer and can affect approximately 40% of breast cancer survivors [4]. The psychosocial adjustment to breast cancer may differ among women, with some patients experiencing improvement and others deterioration over time. Indeed, several studies have reported trajectories of anxiety and depression symptoms. However, these assessments were based on relatively short follow-up periods, mainly up to one year, and, in certain cases, restricted to patients undergoing specific breast cancer treatments, namely chemotherapy or radiotherapy [5,6,7,8,9,10]. Multiple events affect women with breast cancer in the first year after the cancer diagnosis, namely staging exams, decisions about breast cancer surgery, and treatments. However, the following years are accompanied by sequelae and late effects of treatments [11], fear of recurrence and unmet needs [12], financial difficulties [13], returning to work and to social events [14], and transference of cancer care to primary care services [15], which may also contribute to anxiety and depression symptoms.

Therefore, studies with longer follow-ups are needed to understand the occurrence of anxiety and depression symptoms after the critical first year after a cancer diagnosis. This study aims to describe the trajectories of anxiety and depression symptoms levels in a cohort of patients with breast cancer during the first five years after diagnosis and to identify their predictors.

## 2. Materials and Methods

### 2.1. The NEON-BC Cohort

This study is based on the project NEON-BC, which aimed to investigate the neuro-oncological complications of breast cancer and its treatments. This prospective cohort study included women consecutively recruited in 2012 among those with newly diagnosed breast cancer proposed for surgery at the Breast Clinic of the Portuguese Institute of Oncology of Porto (IPO-Porto), Portugal.

The study methodology has been described in detail elsewhere [16]. Briefly, women who did not have a history of chemotherapy or radiotherapy treatment for another primary cancer, had no previous breast surgery, and were able to understand the purpose of the study were invited to participate (588 eligible patients; two refusals). Those who presented a Montreal Cognitive Assessment (MoCA) score lower than 17 (or 16 if they were aged 65 years or more) were excluded because of the high probability of cognitive impairment [17] and low ability to answer self-questionnaires (80 patients excluded).

A total of 506 women were evaluated at baseline, before any treatment for breast cancer, and at one (n = 503), three (n = 475), and five years (n = 466) since breast cancer diagnosis; a total of 464 participants were evaluated in all moments, and 4 had incomplete questionnaires regarding anxiety and depression scores. Among those lost to follow-up, 18 died, 12 abandoned the study, 4 were transferred to another hospital, 2 were considered unable to participate by the neurologist, and 4 could not be contacted. Two participants were evaluated at the five-year evaluation but not at the three years.

### 2.2. Measurements

Sociodemographic characteristics and lifestyles were assessed through interviews using a structured questionnaire. Individual monthly income was collected at baseline (EUR 500 or lower, EUR 501–1000, EUR 1001–1500, EUR 1500–2000, and >EUR 2000). Data on lifestyles (alcohol consumption, smoking, fruit and vegetable intake, and physical activity), and income were obtained at the three- and five-year evaluations; baseline levels of exposure were assessed retrospectively at the three-year assessment. When menopausal status was not specified in the medical records, all women with at least 60 years, women who underwent bilateral oophorectomy, and those with an intact uterus and being amenorrheic for 12 or more consecutive months prior to the diagnosis, in the absence of alternative pathological or physiological cause or follicle-stimulating hormone and serum estradiol levels within the laboratory’s reference ranges, were classified as postmenopausal, or otherwise as premenopausal [18]. The consumption of medicines was self-reported by the participants at baseline and at the five-year evaluation. Drugs were classified as anxiolytics and antidepressants according to the drug classification of the WHO Collaborating Centre for Drug Statistics Methodology [19]. Clinical characteristics, namely breast cancer stage classified according to the American Joint Committee on Cancer Tumor Node Metastases classification 7th edition [20], and treatment details were abstracted from clinical files at baseline and after one, three, and five years of follow-up. Breast cancer subtype was attributed based on information from medical files regarding immunohistochemistry and in situ hybridization-based biomarkers, namely hormone receptors (HR; estrogen and progesterone receptors, considered positive if present in ≥1% of cells) and human epidermal growth factor receptor (HER2), and classified as HR-positive/HER2-negative (HR+/HER2-); HER2-positive (HER2+); and triple-negative breast cancer (HR-negative/HER2-negative).

The Hospital Anxiety and Depression Scale (HADS) [21], validated in the Portuguese population [22], was used to assess anxiety and depression symptoms levels. It is a multiple-choice questionnaire with 14 questions, 7 regarding depression and 7 regarding anxiety. Each item is scored from 0 to 3, and the maximum score for each subscale is 21. Scores of 11 or higher in the depression or anxiety subscales were considered to indicate clinically significant depression and anxiety symptoms, respectively (i.e., probable cases of depression and anxiety, respectively); scoring between 8 and 10 were considered a borderline disorder, and scores bellow 8 were considered normal [21].

### 2.3. Statistical Analysis

We modeled two outcomes: HADS-A and HADS-D scores according to time. Time was measured from entering the cohort to five years later (mean time = 1696.57 days).

We used the R Statistic Software, version 4.0.5 (R Core Team, Vienna, Austria), to identify the trajectories of anxiety and depression scores over time. The mgcv library [23,24,25,26] was used to fit generalized additive-mixed models with integrated smoothness estimation, which allows inserting smoothing terms for a better fitting to the data. These models included random intercepts and time slopes that were extracted to be included as input variables in the cluster analysis. Mclust [27] was used to obtain model-based clusters of the trends in HADS-A and HADS-D scores over the five years. The decision regarding the number of clusters was based on the Bayesian Information Criteria. Graphical representations of the trajectories were obtained using the ggplot2 library [28] (Supplementary Material, Figures S1 and S2).

Subsequent statistical analyses were performed using STATA^®^, version 15.1 (StataCorp, College Station, TX, USA).

We used the Chi-square test to compare patients’ characteristics and clinical variables between groups defined by anxiety trajectories and depression trajectories. The association between variables measured at baseline or during follow-up and the five-year anxiety/depression trajectories was estimated with adjusted odds ratios (aORs), and respective 95% confidence intervals (CI) were computed using multivariable logistic regression. Variables considered as possible confounders according to the literature were included in the logistic models and are listed in the footnotes of the tables.

To ease the interpretation of the results on the associations of age and education with anxiety and depression, age was categorized in <50, 50–64, and ≥65 years, and education in ≤4, 5–9, 10–12, and >12 years. Logistic models with age and education as continuous variables gave essentially the same results as reported in the Results section. According to the self-reported use of anxiolytics and antidepressants at baseline and at five years, participants were classified as never users, ex-users (users at baseline but not at five years), initiators (not users at baseline but users at five years), and persistent users (users at baseline and at five years).

The predictive accuracy of the variables significantly associated with the trajectories-based groups was further assessed using Receiver Operating Characteristic curves [29], and the corresponding areas under the curve (AUC) were compared. Only baseline variables and variables of the first year of follow-up significantly associated with the trajectories-based groups were considered; thus, having job loss or loss of income were not included in the predictive models as they were not assessed before the three-year evaluation. Age and education were inserted as continuous variables (years), and income (above/below EUR 500), fruit and vegetable intake (≥5/<5 pieces per day), cancer-related neuropathic pain (yes/no), anxiety (yes/no), and depression (yes/no), as dichotomous variables.

All tests were two-sided, and a *p* < 0.05 was considered significant.

## 3. Results

### 3.1. Characterization of the Cohort over Time

A total of 460 participants had completed HADS questionnaires at all evaluations, and they were younger (mean, standard deviation (sd), in years: 54.4, 10.7 vs. 58.2, 15.1; *p* = 0.028) than those who were not evaluated in all assessments (n = 44) or had incomplete HADS questionnaires (n = 4), but not significantly different regarding education (mean, sd, in years: 7.7, 4.5 vs. 6.7, 1.4; *p =* 0.154), baseline anxiety and depressive symptoms (mean, sd: 9.3, 4.2 vs. 8.7, 4.7; *p =* 0.340 and, 5.3, 3.7 vs. 5.3, 4.3; *p =* 0.989, respectively).

At baseline, 49.8% of the women had more than 55 years old, and 42.7% had ≤ 4 complete years of formal education. The majority were married or living with a partner and employed (69.8%; 55.5%, respectively). Only 35.8% of all participants did not consume any type of medicines; 19.4% were taking anxiolytics and 18.8% antidepressants. Most of the women had early cancer stage (ductal carcinoma in situ/stage I; 53.5%), and the most frequent subtype was HR+/HER2- (76.4%), followed by HER2+ (15.4%) and triple-negative (8.2%).

During the follow-up, besides breast surgery, all women except 15 received (neo)adjuvant treatments: 59.2% underwent chemotherapy, 73.6% radiotherapy, 83.9% hormone therapy, and 13.3% were treated with trastuzumab. A total of 3.2% had a breast cancer recurrence, and 5.4% were diagnosed with a new primary tumor (Table 1).

### 3.2. Identification of Anxiety and Depression Trajectories

**Figure 1:**
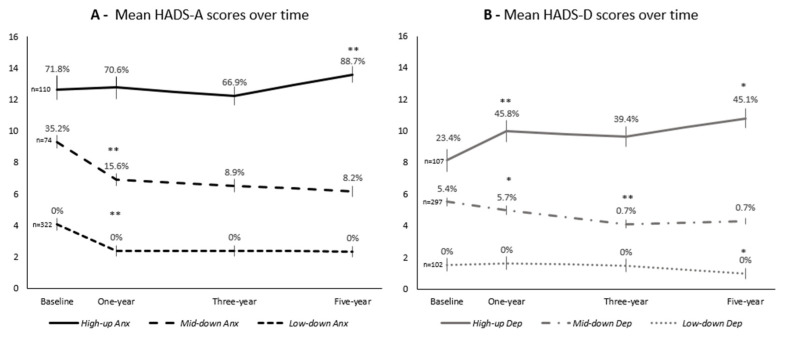
Variation in anxiety (**A**) and depression (**B**) symptoms over the five-year follow-up since breast cancer diagnosis, according to anxiety and depression symptoms trajectories-based groups (*High-up Anx*, *Mid-down Anx*, and *Low-down Anx*, and *High-up Dep*, *Mid-down Dep*, and *Low-down Dep*, respectively). Graphics obtained using Microsoft Excel software. HADS-A—anxiety subscale of the Hospital Anxiety and Depression Scale; HADS-D—depression subscale of the Hospital Anxiety and Depression Scale. Vertical lines represent the 95% confidence interval of mean values. Percentages refer to women with clinically significant symptoms of anxiety (**A**; HADS-A ≥ 11) or depression (**B**; HADS-D≥11). * *p* < 0.05 for comparison of scores between precedent and present evaluation. ** *p* < 0.001 for comparison of scores between precedent and present evaluation.

Three trajectories of anxiety levels were identified (Figure 1A): in the *High-up Anx* trajectory group (n = 110, 21.7% of the cohort), scores were stable over the first three years, and then a significant increase was observed at the five-year evaluation, with an increase in the prevalence of clinically significant anxiety symptoms from 71.8% at baseline to 88.7%. In the *Mid-down Anx* trajectory group (n = 74, 14.6%), a significant decrease in scores was observed at one year, and afterward, a stabilization; 35.2% of women had clinically significant anxiety symptoms at baseline, 15.6% at one year and nearly 8% afterwards; in the *Low-down Anx* group (n = 322, 63.6%), women had normal levels of anxiety symptoms at baseline (0% clinically significant anxiety), decreasing significantly in the first year, and continuing to decrease over time.

The depression trajectories (Figure 1B) can be described as follows: in the *High-up Dep* trajectory group (n = 107, 21.1%), levels of depression significantly increased at one year (45.8% had clinically significant depressive symptoms), then stabilized, and increased significantly again at five years; in the *Mid-down Dep* trajectory group (n = 297, 58.7%), there was a significant decrease in scores after three years of follow-up, and then a stabilization; the *Low-down Dep* trajectory group (n = 102, 20.2%) had the lowest scores over time and no cases of clinically significant depression symptoms.

Most participants (45.5%) were simultaneously in the groups *Mid-down Anx* and *Mid--down Dep*, 13.0% were in the *High-up Anx* and *High-up Dep*, 9.7% were in the *Low-down Anx* and *Low-down Dep*, and the other combinations of anxiety and depression groups included 8-10% of participants, except the combinations of High-up with Low-down with only one participant in each.

Table 2 presents the ORs for the association between variables measured at baseline and during the follow-up, with *High-up Anx* vs. *Mid-down Anx* trajectories and *High-up Dep* vs. *Mid-down Dep* trajectories. Significant associations were observed between baseline variables with *High-up Anx* trajectory, namely, higher age (age ≥ 65 vs. <50 years; adjusted OR [aOR] = 0.30, 95% CI, 0.13–0.70), higher education level (>12 vs. ≤4 years, aOR = 0.41, 95% CI, 0.18–0.96), individual monthly income above EUR 500 (aOR = 0.43, 95% CI, 0.24–0.76), fruit and vegetable consumption equal or higher than five pieces a day (aOR = 0.51, 95% CI, 0.28–0.95), and having clinically significant anxiety and depression symptoms (aOR = 4.99, 95% CI, 3.05–8.15; aOR = 3.26, 95% CI, 1.66–6.40, respectively). Moreover, having cancer-related neuropathic pain during follow-up (aOR = 3.43, 95% CI, 2.15–5.46) and having lost the job (aOR = 3.58, 95% CI, 1.57–8.15) was also associated with *High-up Anx* trajectory. Initiators and persistent users of anxiolytics and antidepressants were more likely to pertain to the worst anxiety trajectory (aORs ranging between 2.41 and 3.58).

The only significant associations between baseline variables with *High-up Dep* trajectory were clinically significant anxiety and depression symptoms (aOR = 2.41, 95% CI, 1.52–3.81; aOR = 5.28, 95% CI, 2.66–10.46, respectively). Moreover, having cancer-related neuropathic pain during follow-up (OR = 2.89, 95% CI, 1.80–4.62), having lost the job (OR = 2.36, 95% CI, 1.01–5.51), and having lost income (OR = 1.77, 95% CI, 1.05–2.98), were also associated with *High-up Dep* trajectory. Initiators and persistent users of anxiolytics and antidepressants were more likely to pertain to the worst depression trajectory (aORs ranging between 3.47 and 4.67).

**Figure 2:**
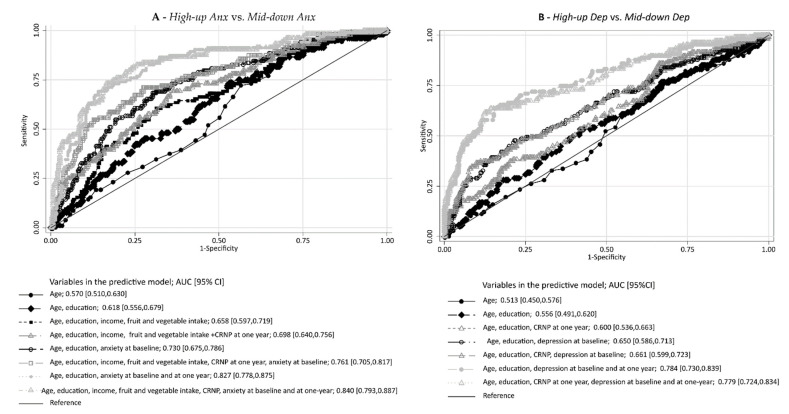
Predictive models for *High-up Anx* vs. *Mid-down Anx* trajectory (**A**) and for *High-up Dep* vs. *Mid-down Dep* trajectory (**B**).

Figure 2A depicts the ROC curves of the predictive models for the classification of *High-up Anx* vs. *Mid-down Anx* trajectories, which included the variables for which significant associations were observed. The best models were those which included the classification of clinically significant anxiety at baseline and at one year. No significant increase in the AUC was observed when income, fruit and vegetable intake, and cancer-related neuropathic pain were added to the model, including age, education, and anxiety at baseline and one year (AUC = 0.827 and AUC = 0.840; *p* = 0.269).

Figure 2B depicts the ROC curves of predictive models for the classification of *High-up Dep* vs. *Mid-down Dep* trajectories. Similarly, the best models were those with the classification of clinically significant depressive symptoms at baseline and at one year, and the inclusion of cancer-related neuropathic pain at one year did not significantly increase the AUC of the model, including age, education, and baseline and one-year depression status (AUC = 0.779 and AUC = 0.784; *p* = 0.625).

Table 3 presents the sensitivity, specificity, positive and negative predictive values, and positive and negative likelihood ratios (LR+ and LR−) of the predictive model, including age, education, and baseline and one-year anxiety status, to predict the *High-up Anx* trajectory, and the predictive model including age, education, and baseline and one-year depression status, to predict the *High-up Dep* trajectory, according to different a priori probabilities of these trajectories. For a priori probabilities above 50% of the *High-up Anx* trajectory, the sensitivity, specificity, positive predictive value, and negative predictive value of the model were: 56.0%, 90.0%, 65.6%, and 85.7%, respectively. The corresponding positive likelihood ratio was 5.6, and the negative likelihood ratio was 0.5.

Similarly, for a priori probabilities above 50% of *High-up Dep* trajectory, the sensitivity, specificity, positive predictive value, and negative predictive value of the predictive model were: 45.8%, 94.3%, 74.2%, and 82.8%, respectively. The corresponding positive likelihood ratio was 8.0 and the negative likelihood ratio 0.6, which correspond to an increase from 50% to nearly 90% of the predicted probability of having a high and increasing trajectory of depressive symptoms over five years and reducing from 50% to 35–40% the predicted probability of having a declining trajectory of depressive symptoms.

## 4. Discussion

Three trajectories of anxiety symptoms and three of depressive symptoms were identified among women with breast cancer followed for five years. The *High-up Anx* and *High-up Dep* trajectories, which correspond to high levels of anxiety and depression symptoms, respectively, during the five years of follow-up, with high scores and an increasing pattern over time, were equally frequent, with nearly one in five women pertaining to each. The other trajectories, two for anxiety and two for depression symptoms, showed a decline in scores over time: one trajectory with mid-range scores and one with low scores for anxiety symptoms, and similarly, one mid-range scores and one low scores trajectory, for depression symptoms. Baseline patients’ characteristics associated with the *High-up Anx* trajectory were identified but not for the *High-up Dep* trajectory. The predictive models, including age, education, and anxiety/depression status at baseline and at one year were the ones with higher AUC, and their ability to predict the worst anxiety and depression trajectories may be considered moderate/large to rule in but small to rule out, considering a pretest probability of 50%.

Several studies have reported anxiety and depression trajectories of breast cancer patients, but the duration of follow-up was up to one year [5,6,7,8,9,10], or two years [30], which precludes direct comparisons with our results. Indeed, although a high scores trajectory of anxiety symptoms was reported in two studies [8,30], no significant variation over time was observed, while in the present study, in the *High-up Anx* trajectory, we observed a clear and significant worsening of anxiety symptoms at the five-year evaluation. At IPO-Porto, many patients may have hospital discharge and start follow-up at primary care services five years after diagnosis, which may have contributed to an increase in anxiety symptoms [15].

Our results for depression trajectories are relatively different from the ones found in previous studies with a follow-up not longer than one year [5,6,7,9,10]. In general, at least three depression trajectories with the declining pattern were described, and no trajectory of increasing levels of depression, except in one study which describes four trajectories over six months since breast surgery, with one presenting a late increase [9]. Two studies focused on the chemotherapy treatment period [7,10], and the results may not be generalized to patients not treated with chemotherapy. Another study was based on two clinical trials with interventions to increase the quality of life [6], which could also change the course of depression symptoms. Therefore, the present study presents new information on anxiety and depression trajectories in a cohort with different treatments and a long follow-up of five years.

In the NEON-BC cohort, participants 65 years of age or older were less likely to present the worst anxiety trajectory. Another study also identified an association between younger age (<50 years) and higher anxiety scores (in the HADS) after 5–6 years since breast cancer diagnosis [31]. Higher education level was a protective factor regarding the worst anxiety trajectory in our study, which may be explained by the better access and comprehension of clinical information on cancer diagnosis and treatments [32]. We also observed that fruit and vegetable intake of at least five portions per day was associated with a declining trajectory of anxiety symptoms rather than an increasing trajectory. This may be explained by the lower oxidative stress conferred by fruit and vegetables, which was associated with lower anxiety levels in a previous study [33]. We also identified that lower income at baseline was associated with the worst trajectory of anxiety symptoms. Although in Portugal, patients have access to cancer care as part of the universal health coverage, patients with low income may experience financial distress due to transportation, parking costs, and loss of job [13], which could contribute to anxiety scores remaining high.

Initiators and persistent users of anxiolytics and antidepressants were more likely to pertain to the worst anxiety and depression trajectories, which suggests that these medicines may not be as effective as desired. However, we did not collect information on the use of medicines at one and three years and on non-pharmacological treatments for anxiety and depression, which may also have influenced the trajectories over time.

In our study, cancer-related neuropathic pain was strongly associated with the worse anxiety and depression symptoms trajectories. This may be explained by the catastrophizing phenomenon (negative pain-related cognitions that obstruct the ability to deal with pain and tend to exaggerate its value), which have been shown to be one of the most significant determinants of depression and anxiety in patients with neuropathic pain [34]. Although we identified baseline factors associated with the worst trajectory of anxiety symptoms, we could not identify baseline determinants of the trajectory with high and increasing levels of depression, except for the presence of clinically significant anxiety and depression symptoms at baseline. In-depth studies of the patients’ characteristics could reveal other determinants of the worse depression trajectory, for instance, fear of receiving compassion from others [35] and coping and optimism [36], which have been associated with depression. Nevertheless, we showed that predictive models that include basic data of the patients, age, education, and the scores in the HADS before treatment and after one year may be useful to predict this long-term trajectory. Therefore, the use of the HADS during the first year after cancer diagnosis could identify patients who currently need psychological support and those who would benefit from further assessments over the next five years.

This study benefits from the low attrition rate of the NEON-BC cohort (7.9% over the five years of follow-up), its sample size, and the long follow-up of five years since cancer diagnosis, which allowed to identify the long-term trajectories of anxiety and depression symptoms. However, the NEON-BC cohort included mostly women with early-stage breast cancer, and therefore our results may not be generalized to patients with advanced breast cancer. Moreover, only one hospital took part in the study, although IPO-Porto represents the largest cancer hospital in the Northern region of Portugal, which admits patients after a referral from the family doctor or according to interhospital collaboration protocols.

## 5. Conclusions

The present study shows that persistent and high levels of anxiety and depression symptoms affected one-fifth of the women with breast cancer during the first five years following a cancer diagnosis. These long-term trajectories were largely predicted by baseline and one-year status of probable anxiety and depression. These results show that assessing anxiety and depression symptoms before treatment and after one year may contribute to identifying the patients who could benefit the most from psychological support.

## Figures and Tables

**Table 1:** Sociodemographic characteristics of participants, lifestyles, medicines consumption, breast tumor characteristics, and treatments, according to anxiety and depression trajectories.

	All	*High-Up Anxiety*	*Mid-Down Anxiety*	*Low-Down Anxiety*	*χ* ^2^ *Test*	*High-Up* *Depression*	*Mid-Down Depression*	*Low-Down Depression*	*χ*^2^ Test
	n (%)	n (%)	n (%)	n (%)	*p*	n (%)	n (%)	n (%)	*p*
At baseline									
Socio-demographics									
Age (years)					<0.001				0.227
<50	178 (35.2)	40 (36.4)	115 (35.7)	23 (31.1)		35 (32.7)	99 (33.3)	44 (43.1)	
50–64	230 (45.4)	63 (57.3)	139 (43.2)	28 (37.8)		54 (50.5)	133 (44.8)	43 (42.2)	
≥65	98 (19.4)	7 (6.4)	68 (21.1)	23 (31.1)		18 (16.8)	65 (21.9)	15 (14.7)	
Education (years)					0.409				0.354
≤4	216 (42.7)	46 (41.8)	140 (43.5)	30 (40.5)		46 (43.0)	131 (44.1)	39 (38.2)	
5–9	145 (28.7)	37 (33.6)	89 (27.6)	19 (25.7)		37 (34.6)	81 (27.3)	27 (26.5)	
10–12	78 (15.4)	19 (17.3)	47 (14.6)	12 (16.2)		15 (14.0)	46 (15.5)	17 (16.7)	
>12	67 (13.2)	8 (7.3)	46 (14.3)	13 (17.6)		9 (8.4)	39 (13.1)	19 (18.6)	
Postmenopausal	288 (56.9)	59 (53.6)	180 (55.9)	49 (66.2)	0.199	62 (57.9)	173 (58.2)	53 (52.0)	0.527
Marital status					0.457				0.425
Married/living with partner	353 (69.8)	82 (74.5)	225 (69.9)	46 (62.2)		70 (65.4)	217 (73.1)	66 (64.7)	
Single	53 (10.5)	9 (8.2)	33 (10.2)	11 (14.9)		12 (11.2)	28 (9.4)	13 (12.7)	
Widow/divorced	100 (19.8)	19 (17.3)	64 (19.9)	17 (23.0)		25 (23.4)	52 (17.5)	23 (22.5)	
Employed	279 (55.5)	65 (59.1)	180 (56.3)	34 (46.6)	0.223	51 (48.1)	171 (57.8)	57 (56.4)	0.224
Individual monthly income >EUR500	210 (45.0)	34 (32.7)	143 (47.8)	33 (51.6)	0.015	36 (35.3)	122 (45.0)	52 (55.3)	0.019
Living in Great Porto area	226 (44.7)	47 (42.7)	142 (44.1)	37 (50.0)	0.588	45 (42.1)	133 (44.8)	48 (47.1)	0.766
Lifestyles									
Daily alcohol consumption >10 g	97 (19.2)	16 (14.7)	60 (18.6)	21 (28.4)	0.063	19 (17.9)	63 (21.2)	15 (14.7)	0.331
Ever smoker	107 (23.1)	30 (28.3)	61 (20.7)	16 (25.0)	0.264	22 (21.6)	63 (23.5)	22 (23.4)	0.921
Fruit and vegetables consumption ≥5 portions/day	104 (22.0)	15 (14.2)	74 (24.5)	15 (23.4)	0.083	17 (16.5)	66 (24.0)	21 (22.3)	0.293
Medicines consumption					0.656				0.053
None	181 (35.8)	38 (34.5)	110 (34.2)	33 (44.6)		35 (32.7)	107 (36.0)	39 (38.2)	
1–2	85 (16.8)	22 (20.0)	52 (16.1)	11 (14.9)		14 (13.1)	48 (16.2)	23 (22.5)	
3–4	161 (31.8)	34 (30.9)	106 (32.9)	21 (28.4)		39 (36.4)	88 (29.6)	34 (33.3)	
≥5	79 (15.6)	16 (14.5)	54 (16.8)	9 (12.2)		35 (32.7)	107 (36.0)	39 (38.2)	
Anxiolytics	98 (19.4)	30 (27.3)	61 (18.9)	7 (9.5)	0.011	34 (31.8)	51 (17.2)	13 (12.8)	0.001
Antidepressants	95 (18.8)	28 (25.4)	61 (18.9)	6 (8.1)	0.013	34 (31.8)	52 (17.5)	9 (8.8)	<0.001
Breast cancer characteristics									
Cancer stage					0.489				0.701
0/I	270 (53.5)	61 (55.5)	176 (54.7)	33 (45.2)		56 (52.3)	161 (54.2)	53 (52.5)	
II	156 (30.9)	31 (28.2)	98 (30.4)	27 (37.0)		32 (29.9)	91 (30.6)	33 (32.7)	
III	75 (14.9)	18 (16.4)	44 (13.7)	13 (17.8)		19 (17.8)	43 (14.5)	13 (12.9)	
IV	4 (0.8)	0 (0.0)	4 (1.2)	0 (0.0)		0 (0.0)	2 (0.7)	2 (2.0)	
Subtypes					0.380				0.333
HR+/HER2-	362 (76.4)	82 (79.6)	233 (77.4)	47 (67.1)		84 (84.0)	208 (75.1)	70 (72.2)	
HER2+	73 (15.4)	13 (12.6)	45 (15.0)	15 (21.4)		11 (11.0)	44 (15.9)	18 (18.6)	
Triple-negative	39 (8.2)	8 (7.8)	23 (7.6)	8 (11.4)		5 (5.0)	25 (9.0)	9 (9.3)	
During follow-up									
Treatments									
Breast surgery					0.744				0.379
Breast-conserving surgery	250 (49.6)	51 (46.4)	162 (50.5)	37 (50.7)		47 (43.9)	150 (50.5)	53 (53.0)	
Mastectomy	254 (50.4)	59 (53.6)	159 (49.5)	36 (49.3)		60 (56.1)	147 (49.5)	47 (47.0)	
Axillary Surgery	174 (35.5)	39 (36.1)	106 (34.1)	29 (40.8)	0.555	36 (35.3)	100 (34.4)	38 (39.2)	0.691
Chemotherapy	298 (59.2)	63 (57.8)	189 (58.9)	46 (63.0)	0.763	60 (56.1)	170 (57.4)	68 (68.0)	0.134
Radiotherapy	370 (73.6)	75 (68.8)	236 (73.5)	59 (80.8)	0.197	75 (70.1)	217 (73.3)	78 (78.0)	0.431
Endocrine therapy	422 (83.9)	88 (80.7)	271 (84.4)	63 (86.3)	0.553	90 (84.1)	244 (82.4)	88 (88.0)	0.423
Trastuzumab	67 (13.3)	12 (11.0)	42 (13.1)	13 (17.8)	0.408	11 (10.3)	39 (13.2)	17 (17.0)	0.362
Recurrence	15 (3.2)	1 (0.9)	11 (3.7)	3 (4.6)	0.303	3 (2.9)	8 (3.0)	4 (4.2)	0.830
New primary tumor	25 (5.4)	9 (8.5)	13 (4.4)	3 (4.7)	0.263	9 (8.8)	13 (4.8)	3 (3.2)	0.178
Changes in sociodemographic characteristics									
Became widow/divorced/separated	39 (7.8)	4 (3.6)	26 (8.1)	9 (12.3)	0.090	5 (4.7)	27 (9.1)	7 (7.0)	0.321
Loss of job	29 (5.7)	15 (13.6)	11 (3.4)	3 (4.1)	<0.001	11 (10.3)	13 (4.4)	5 (4.9)	0.073
Became retired	41 (8.1)	10 (9.1)	27 (8.4)	4 (5.4)	0.637	8 (7.5)	29 (9.8)	4 (3.9)	0.169
Loss of income	113 (22.3)	32 (29.1)	65 (20.2)	16 (21.6)	0.152	32 (29.9)	58 (19.5)	23 (22.5)	0.087
Use of anxiolytics over time					<0.001				<0.001
Never users ^a^	286 (61.4)	43 (40.6)	189 (63.9)	54 (84.4)		34 (33.3)	179 (66.3)	73 (77.7)	
Ex-users ^b^	25 (5.4)	5 (4.7)	16 (5.4)	4 (6.2)		5 (4.9)	17 (6.3)	3 (3.2)	
Initiators ^c^	87 (18.7)	34 (32.1)	50 (16.9)	3 (4.7)		36 (35.3)	43 (15.9)	8 (8.5)	
Persistent users ^d^	68 (14.6)	24 (22.6)	41 (13.9)	3 (4.7)		27 (26.5)	31 (11.5)	10 (10.6)	
Use of antidepressants over time					<0.001				<0.001
Never users ^a^	304 (65.2)	48 (45.3)	201 (67.9)	55 (85.9)		41 (40.2)	183 (67.8)	80 (85.1)	
Ex-users ^b^	27 (5.8)	6 (5.7)	19 (6.4)	2 (3.1)		6 (5.9)	16 (5.9)	5 (5.3)	
Initiators ^c^	71 (15.2)	30 (28.3)	37 (12.5)	4 (6.2)		28 (27.4)	38 (14.1)	5 (5.3)	
Persistent users ^d^	64 (13.7)	22 (20.8)	39 (13.2)	3 (4.7)		27 (26.5)	33 (12.2)	4 (4.3)	

HR+/HER2-, estrogen and progesterone receptors positive and human epidermal growth factor receptor (HER2) negative; HER2+, human epidermal growth factor receptor positive. ^a^ never users are participants who did not report the use of this class of medicines neither at baseline nor at five years; ^b^ ex-users are participants who reported using this class of medicines at baseline but not at five years; ^c^ initiators are participants who reported using this class of medicines at five years but not at baseline; ^d^ persistent users are participants who reported using this class of medicines at baseline and at five years.

**Table 2:** Associations between baseline and follow-up variables with *High-up Anx* vs. *Mid-down Anx* trajectory and with *High-up Dep* vs. *Mid-down Dep* trajectory.

	*High-Up Anx* vs. *Mid-Down Anx*		*High-Up Dep* vs. *Mid-Down Dep*		
	OR	[95% CI]	OR	[95% CI]	
Baseline Variables					
Age (years) 50–64 vs. <50	1.30	[0.82,2.08]	1.15	[0.70,1.89]	
Age (years) ≥ 65 vs. <50	0.30	[0.13,0.70]	0.78	[0.41,1.50]	
Education (years) 5–9 vs. ≤4	1.01	[0.59,1.75]	1.23	[0.70,2.15]	^a^
Education (years) 10–12 vs. ≤4	1.01	[0.52,1.96]	0.89	[0.44,1.80]	^a^
Education (years) >12 vs. ≤4	0.41	[0.18,0.96]	0.62	[0.27,1.42]	^a^
Post-menopausal vs. pre-menopausal	1.04	[0.50,2.15]	0.97	[0.45,2.11]	^b^
Single vs. married/living with partner	0.83	[0.37,1.83]	1.41	[0.67,2.94]	^b^
Widow/Divorced vs. married/living with partner	1.06	[0.58,1.93]	1.66	[0.94,2.92]	^b^
Employed vs. unemployed	0.97	[0.58,1.61]	0.60	[0.36,1.01]	^b^
Individual monthly income > EUR500 vs. ≤ EUR500	0.43	[0.24,0.76]	0.64	[0.36,1.14]	^b^
Daily alcohol consumption >10 g vs. ≤10 g	0.71	[0.38,1.32]	0.77	[0.43,1.38]	^b^
Ever smoker vs. never smoker	1.51	[0.87,2.65]	0.90	[0.50,1.64]	^b^
Fruit and vegetables consumption ≥5/day vs. <5/day	0.51	[0.28,0.95]	0.64	[0.36,1.17]	^b^
Practicing physical activity vs. not	0.66	[0.33,1.33]	0.70	[0.35,1.40]	^b^
Cancer stage II vs. 0/I	0.88	[0.52,1.47]	1.00	[0.59,1.67]	^b^
Cancer stage III/IV vs. 0/I	1.01	[0.54,1.91]	1.18	[0.63,2.21]	^b^
Subtype HER2+ vs. HR+/HER2-	0.74	[0.38,1.47]	0.59	[0.29,1.21]	^b^
Subtype Triple negative vs. HR+/HER2-	0.85	[0.36,2.01]	0.47	[0.17,1.28]	^b^
Clinically significant anxiety symptoms vs. not	4.99	[3.05,8.15]	2.41	[1.52,3.81]	^b^
Clinically significant depression symptoms vs. not	3.26	[1.66,6.40]	5.28	[2.66,10.46]	^b^
Variables at follow-up					
Mastectomy vs. breast-conserving surgery	1.33	[0.84,2.08]	1.38	[0.88,2.18]	^c^
Axillary surgery vs. lymph node dissection	1.00	[0.62,1.60]	1.00	[0.62,1.63]	^c^
Chemotherapy vs. no chemotherapy	0.75	[0.47,1.21]	0.85	[0.52,1.38]	^c^
Radiotherapy vs. no radiotherapy	0.62	[0.38,1.03]	0.78	[0.47,1.29]	^d^
Endocrine therapy vs. no endocrine therapy	0.81	[0.46,1.45]	1.14	[0.63,2.09]	^c^
Trastuzumab vs. no Trastuzumab	0.75	[0.38,1.51]	0.72	[0.35,1.48]	^c^
Cancer-related neuropathic pain at any evaluation vs. never	3.43	[2.15,5.46]	2.89	[1.80,4.62]	^b^
Recurrence	0.26	[0.03,2.09]	1.04	[0.27,4.06]	^b^
New primary tumor	2.12	[0.85,5.29]	1.87	[0.76,4.55]	^b^
Became widow/divorced/separated	1.52	[0.71,3.23]	1.21	[0.57,2.57]	^b^
Loss of job vs. still employed	3.58	[1.57,8.15]	2.36	[1.01,5.51]	^b^
Became retired vs. still employed	0.85	[0.39,1.84]	0.64	[0.28,1.47]	^b^
Loss of income	1.45	[0.87,2.41]	1.77	[1.05,2.98]	^b^
Ex-users of anxiolytics vs. never users	1.34	[0.45,3.96]	1.52	[0.51,4.46]	^b^
Initiators of anxiolytics vs. never users	3.56	[1.99,6.34]	4.67	[2.58,8.44]	^b^
Persistent users of anxiolytics vs. never users	2.62	[1.41,4.87]	4.66	[2.45,8.86]	^b^
Ex-users of antidepressants vs. never users	1.12	[0.42,3.00]	1.46	[0.53,3.99]	^b^
Initiators of antidepressants vs. never users	3.58	[1.98,6.49]	3.47	[1.89,6.35]	^b^
Persistent users of antidepressants vs. never users	2.41	[1.27,4.57]	3.94	[2.08,7.44]	^b^

^a^ Adjusted for age; ^b^ adjusted for age and education; ^c^ adjusted for age, education, cancer stage and subtype; ^d^ adjusted for age, education, cancer stage and subtype, breast surgery and axillary surgery.

**Table 3:** Sensitivity, specificity, positive and negative predictive values (PPV and NPV), and positive and negative likelihood ratios (LR+ and LR−) of the predictive model, including age, education, and baseline and one-year anxiety status to predict the *High-up Anx* vs. *Mid-down* trajectories and the predictive model including age, education, and baseline and one-year depression status to predict the *High-up Dep* vs. *Mid-down Dep* trajectories, according to different a priori probabilities (Pr) of these trajectories.

	*High-Up Anx* vs. *Mid-Down Anx*						*High-Up Dep* vs. *Mid-Down Dep*					
Pr %	Sensitivity (%)	Specificity (%)	PPV (%)	NPV (%)	LR+	LR−	Sensitivity (%)	Specificity (%)	PPV (%)	NPV (%)	LR+	LR−
10	90.8	53.4	39.9	94.5	2.0	0.2	98.1	5.7	27.3	89.5	1.0	0.3
20	74.3	80.0	55.9	90.1	3.7	0.3	57.9	87.5	62.6	85.2	4.6	0.5
30	70.6	84.4	60.6	89.4	4.5	0.3	54.2	88.9	63.7	84.3	4.9	0.5
40	67.9	86.9	63.8	88.8	5.2	0.4	51.4	90.5	66.3	83.8	5.4	0.5
50	56.0	90.0	65.6	85.7	5.6	0.5	45.8	94.3	74.2	82.8	8.0	0.6
60	50.5	91.6	67.1	84.4	6.0	0.5	42.1	94.9	75.0	81.9	8.3	0.6
70	23.9	96.9	72.2	78.9	7.6	0.8	30.8	96.0	73.3	79.3	7.6	0.7
80	0	100	-	74.6	-	1	15.0	100.0	100.0	76.5	-	0.8
90	0	100	-	74.6	-	1	9.4	100.0	100.0	75.3	-	0.9

## Data Availability

The datasets generated and analyzed in this study will not be publicly available, given that the included patients do not specifically provide their consent for public sharing of their data and that anonymization is unlikely to be feasible since the identification of patients treated in only one institution within a relatively short period may be possible when taking sociodemographic and clinical characteristics into account.

## Supplementary Materials

The following supporting information can be downloaded at: https://www.mdpi.com/article/10.3390/healthcare10040661/s1, Figure S1: Individual and model-based trajectories of anxiety scores attained in the Hospital Anxiety and Depression Scale, over the five years of follow-up.; Figure S2: Individual and model-based trajectories of depression scores attained in the Hospital Anxiety and Depression Scale, over the five years of follow-up.
