# Supplementary material for: Trajectories of Anxiety and Depression Symptoms over Five Years since Breast Cancer Diagnosis: Results of the NEON-BC Prospective Study
Source: Healthcare (Basel). 2022 Mar 31;10(4):661. doi: 10.3390/healthcare10040661 (PMC9026220; doi:10.3390/healthcare10040661)
Supplement: Supplementary file 1 [file healthcare-10-00661-s001.zip › healthcare-1628188-supplementary.pdf]

Article “Trajectories of anxiety and depression symptoms over five years since breast cancer diagnosis: Results of the NEON-BC prospective study.”

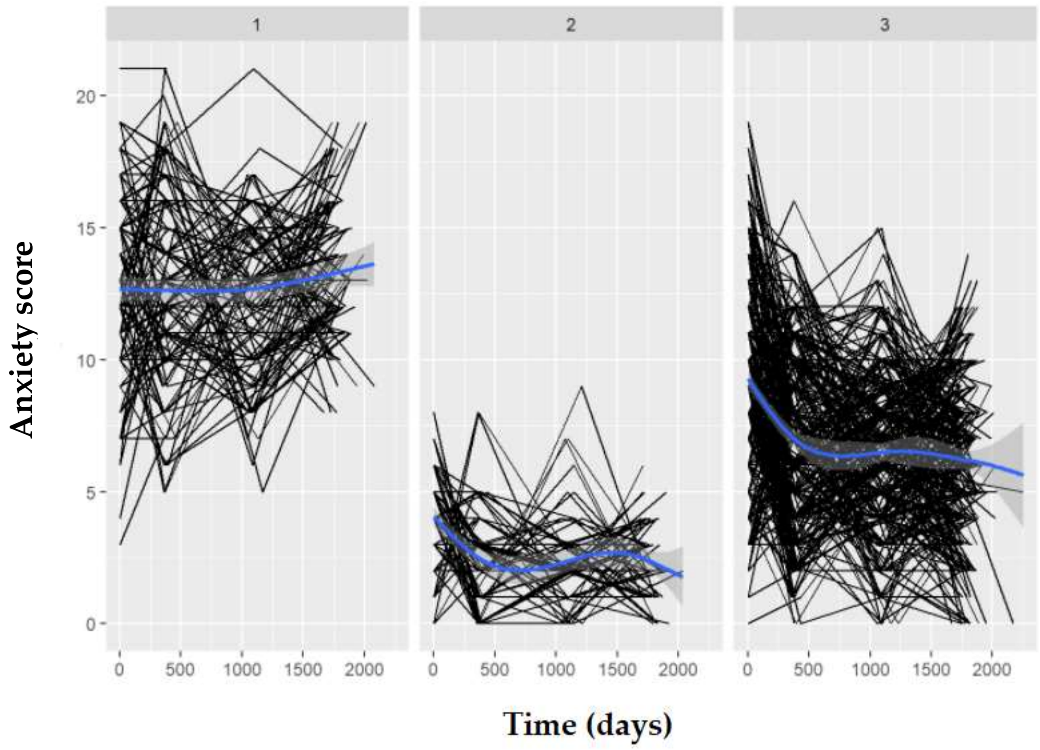

Figure S1. Individual and model-based trajectories of anxiety scores attained in the Hospital Anxiety and Depression Scale, over the five years of follow-up.

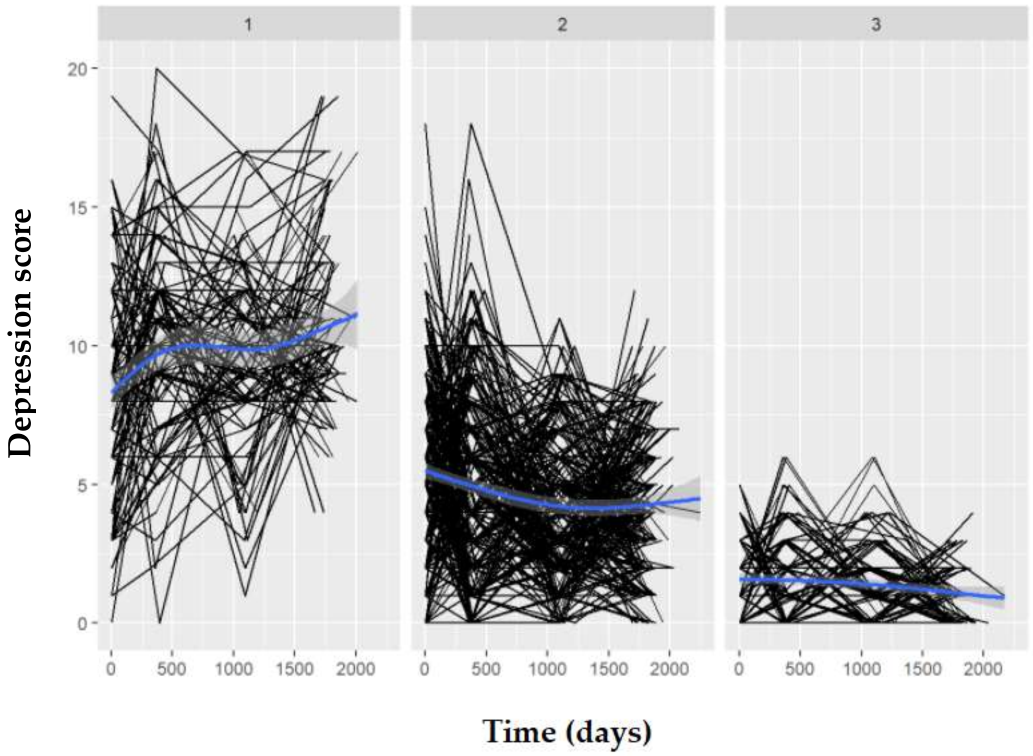

Figure S2. Individual and model-based trajectories of depression scores attained in the Hospital Anxiety and Depression Scale, over the five years of follow-up.
